# Better Progression-Free Survival in Elderly Patients with Stage IV Lung Adenocarcinoma Harboring Uncommon Epidermal Growth Factor Receptor Mutations Treated with the First-line Tyrosine Kinase Inhibitors

**DOI:** 10.3390/cancers10110434

**Published:** 2018-11-13

**Authors:** Ming-Ju Tsai, Jen-Yu Hung, Mei-Hsuan Lee, Chia-Yu Kuo, Yu-Chen Tsai, Ying-Ming Tsai, Ta-Chih Liu, Chih-Jen Yang, Ming-Shyan Huang, Inn-Wen Chong

**Affiliations:** 1 Division of Pulmonary and Critical Care Medicine, Department of Internal Medicine, Kaohsiung Medical University Hospital, Kaohsiung Medical University, No.100, Tz-You 1st Road, Kaohsiung 807, Taiwan; 960216kmuh@gmail.com (M.-J.T.); jenyuhung@gmail.com (J.-Y.H.); mhsuan99@gmail.com (M.-H.L.); goba2356@gmail.com (C.-Y.K.); 1010362kmuh@gmail.com (Y.-C.T.); yingming@kmu.edu.tw (Y.-M.T.); chong@cc.kmu.edu.tw (I.-W.C.); 2 Department of Internal Medicine, Kaohsiung Municipal Ta-Tung Hospital, Kaohsiung 80145, Taiwan; 3 Department of Internal Medicine, School of Medicine, College of Medicine, Kaohsiung Medical University, Kaohsiung 80708, Taiwan; d730093@cc.kmu.edu.tw; 4 Department of Respiratory Care, College of Medicine, Kaohsiung Medical University, Kaohsiung 80708, Taiwan; 5 Graduate Institute of Clinical Medicine, College of Medicine, Kaohsiung Medical University, Kaohsiung 80708, Taiwan; 6 Graduate Institute of Medicine, College of Medicine, Kaohsiung Medical University, Kaohsiung 80708, Taiwan; 7 Division of Hematology and Oncology, Department of Internal Medicine, Kaohsiung Medical University Hospital, Kaohsiung Medical University, Kaohsiung 80708, Taiwan; 8 E-DA Cancer Hospital, Kaohsiung 82445, Taiwan; shyang@kmu.edu.tw; 9 School of Medicine, I-Shou University, Kaohsiung 82445, Taiwan

**Keywords:** lung cancer, adenocarcinoma, gefitinib, erlotinib, afatinib, elderly, epidermal growth factor receptor, tyrosine kinase inhibitor

## Abstract

Patients with lung adenocarcinoma harboring common epidermal growth factor receptor (*EGFR*) mutations usually have a good response rate (RR) and longer progression-free survival (PFS) to *EGFR* tyrosine kinase inhibitors (TKIs). However, the treatment efficacy to uncommon *EGFR* mutations remains controversial. We, therefore, performed a retrospective study, screening 2958 patients. A total of 67 patients with lung adenocarcinoma harboring uncommon *EGFR* mutations were enrolled and 57 patients with stage IV diseases receiving a first-line *EGFR* TKI were included for further analyses. The patients were classified into 27 (47%) “a single sensitizing uncommon mutation”, 7 (12%) “multiple sensitizing mutations”, 5 (9%) “a sensitizing mutation and a resistant uncommon mutation”, and 18 (32%) “other resistant uncommon mutations”. No significant difference was noted in PFS or overall survival (OS) between groups. Patients receiving different first-line *EGFR* TKIs had similar PFS and OS. The elder patients had a significantly poorer performance status than the younger patients but a significantly longer PFS than the younger patients (median PFS: 10.5 vs. 5.5 months, *p* = 0.0320). In conclusion, this is the first study to identify that elderly patients with stage IV lung adenocarcinoma harboring uncommon *EGFR* mutation might have a longer PFS. Large-scale prospective studies are mandatory to prove our findings.

## 1. Introduction

Lung cancer is a leading cause of death in the world. Platinum-based chemotherapy had been a standard therapy for metastatic lung cancer, but the treatment result is still limited and disappointing. In the past decade, non-small cell lung cancer (NSCLC) patients had been proved to have longer progression-free survival (PFS) and better response rate to epidermal growth factor receptor (*EGFR*) tyrosine kinase inhibitors (TKIs) if they a had sensitizing *EGFR* mutation in phase 3 clinical trials [1,2,3,4,5]. Therefore, *EGFR* TKIs had been the standard therapy in NSCLC patients with *EGFR* mutation. Of all *EGFR* mutations in lung cancer, approximately 90% are common mutations, including in-frame deletions in exon 19 and an L858R point mutation in exon 21 [5,6,7]. However, many uncommon mutations, also called rare or non-classical mutations, were detected but the response to *EGFR* TKIs was inconsistent due to a limited number of cases enrolled in the trials [8,9,10,11,12,13,14]. For example, Chiu et al. claimed that both gefitinib and erlotinib are active in lung adenocarcinoma patients with G719X/L861Q/S768I mutations but they had short PFS (a median of 7.7 months) than in those with common *EGFR* mutations (a median of 11.4 months) (*p* < 0.01) [13]. A post-hoc analysis of the LUX-Lung 2, 3 and 6 trials demonstrated that patients harboring G719X, L861G, or S768I mutation responded to afatinib [15]. In a retrospective study by Shen et al., afatinib provided significantly better PFS than gefitinib/erlotinib in 51 patients with stage IIIB-IV lung adenocarcinoma with non-classical mutations (median PFS: 11.0 vs. 3.6 months, *p* = 0.03) [9]. Tu et al. indicated that non-classical mutations were more common in smokers (30.7% vs. 24.3%, *p* = 0.039) and males (54.1% vs. 44.4%, *p* = 0.007), and favorable efficacy was observed in patients harboring L858R mutation (median PFS: 15.2 months) or G719X mutation (median PFS: 11.6 months) [8].

Identifying the predicting factors for the clinical efficacy of *EGFR* TKIs in these patients with lung adenocarcinoma harboring uncommon *EGFR* mutation is urgent. We, therefore, conducted a retrospective study in two university-affiliated hospitals. We made a comprehensive analysis of the clinical efficacy of three different *EGFR* TKIs in these patients.

## 2. Patients and Methods

### 2.1. Patient Identification

In this retrospective study, all patients of lung adenocarcinoma diagnosed and received *EGFR* mutation exam in two university-affiliated hospitals in Taiwan between January 2009 and March 2018 were screened. All patients had uncommon *EGFR* mutation and received an *EGFR* TKI were enrolled and followed until March 2018. The diagnosis of lung cancer was confirmed pathologically, according to World Health Organization pathology classification. The tumor staging was designated according to the seventh American Joint Committee on Cancer staging system by a special committee constituted of clinical pulmonologists, medical oncologists, chest surgeons, radiologists, pathologists and radiation oncologists. Patients were included if: (1) they had adequate tumor specimens for *EGFR* mutation examination, (2) the exam revealed an uncommon *EGFR* gene mutation, and (3) they received an *EGFR* TKI treatment with gefitinib, erlotinib, or afatinib. To clearly identify the effects of *EGFR* TKI as the first-line treatment for stage IV lung adenocarcinoma harboring uncommon mutation, patients with stage I-III cancer and those received an *EGFR* TKI after other treatment modalities were excluded from the further analyses.

As in our previous reports [16,17,18,19,20,21], mutations in *EGFR* gene were analyzed using protocol developed and validated by the Division of Molecular Diagnostics, Department of Laboratory Medicine, Kaohsiung Medical University Hospital (KMUH), which utilized amplification refractory mutation specific (ARMS) polymerase chain reaction (PCR) and Scorpion technologies for the detection, and direct sequencing was performed when a negative result was found in ARMS PCR.

Although a few studies have discussed rare *EGFR* mutation (Table A1), the most appropriate method for classifying rare *EGFR* mutation remained inconclusive because of the rare entity. Therefore, in addition to classifying the mutation patterns by exons, we also classified the mutations by drug sensitivity, including “single sensitizing uncommon mutation” (exon18 G719X and exon21 L861Q), “multiple sensitizing mutations” (exon18 G719X + exon20 S768I, exon18 G719X + exon21 L861Q, and exon21 L858R + exon20 S768I), “a sensitizing mutation and a resistant uncommon mutation” (exon18 G719X + exon18 other and exon21 L858R + exon20 Q812*), and “other resistant uncommon mutations” (exon 18 other mutation, exon 20 insertion, and exon 20 point mutation).

Baseline clinical characteristics were collected by retrospective chart review, including age at diagnosis, sex, initial Eastern Cooperative Oncology Group (ECOG) performance status, smoking history. Smoking status was categorized as current smoker or never smoker (<100 lifetime cigarettes).

Based on serial imaging studies, the Initial treatment response was classified as a progressive disease (PD), stable disease (SD), partial response (PR), or complete response (CR), using the RECIST 1.1 criteria. The primary outcome of this study was PFS on an *EGFR* TKI, defined by the duration between the start of an *EGFR* TKI and the onset of progression under the treatment. The secondary outcome was overall survival (OS), defined by the duration between the date of confirmed diagnosis and the date of death. The study protocol was approved by the KMUH Institutional Review Board (KMUHIRB-E(II)-20150162).

### 2.2. Statistical Analyses

Continuous variables and categorical variables were compared using the Kruskal-Wallis test and Fisher’s exact test, respectively. Survival times, including PFS and OS, were estimated using the Kaplan-Meier method, and differences between the groups were compared using the log-rank test. Cox proportional hazards regression analyses were used to identify the effect of different variables on PFS or OS. After univariate analyses, all variables were included to obtain a maximal model of multivariable analysis to assess the independent effect of different variables. We also used a backward variable selection method, keeping only variables with a *p* value less than 0.15, to develop reduced multivariable models. All statistical analyses were performed using the SAS system (version 9.4 for Windows, SAS Institute Inc., Cary, NC, USA). The statistical significance level was set at a two-sided *p* value of <0.05.

## 3. Results

### 3.1. Patients Characteristics

In 2958 patients with lung adenocarcinoma having their specimens tested for *EGFR* mutation, a total of 67 patients with lung adenocarcinoma harboring uncommon *EGFR* gene mutations, who had received an *EGFR* TKI treatment, were enrolled. Five patients with stage I to III disease (Table A2) were excluded from further analyses to control the confounding effects from the cancer stage. In the 62 patients with stage IV lung cancer (Table 1), 36 (58%) were female and 26 (42%) were male, and the mean age was 65 years. Most of them were never smokers (71%) and had a performance status of ECOG ≤ 1 (71%). All patients had their tumor positive for thyroid transcription factor 1 (TTF-1), except for a patient whose tumor was not tested. The overall disease control rate on an *EGFR* TKI was 73%, and the median PFS on an *EGFR* TKI was 7.5 months (Table 1). 

In the patients with stage IV diseases, 57 (92%) patients received an *EGFR* TKI as their first-line treatment. In these patients, similarly, 32 (56%) were female and 25 (44%) were male, and the mean age was 66 years. Most of them were never smokers (70%) and had a performance status of ECOG ≤ 1 (68%). To avoid bias from various preceding lines of treatment, only patients receiving an *EGFR* TKI as the first-line treatment were included for further survival analyses.

### 3.2. Various Outcomes Related to Different EGFR Mutation Patterns

**Figure 1:**
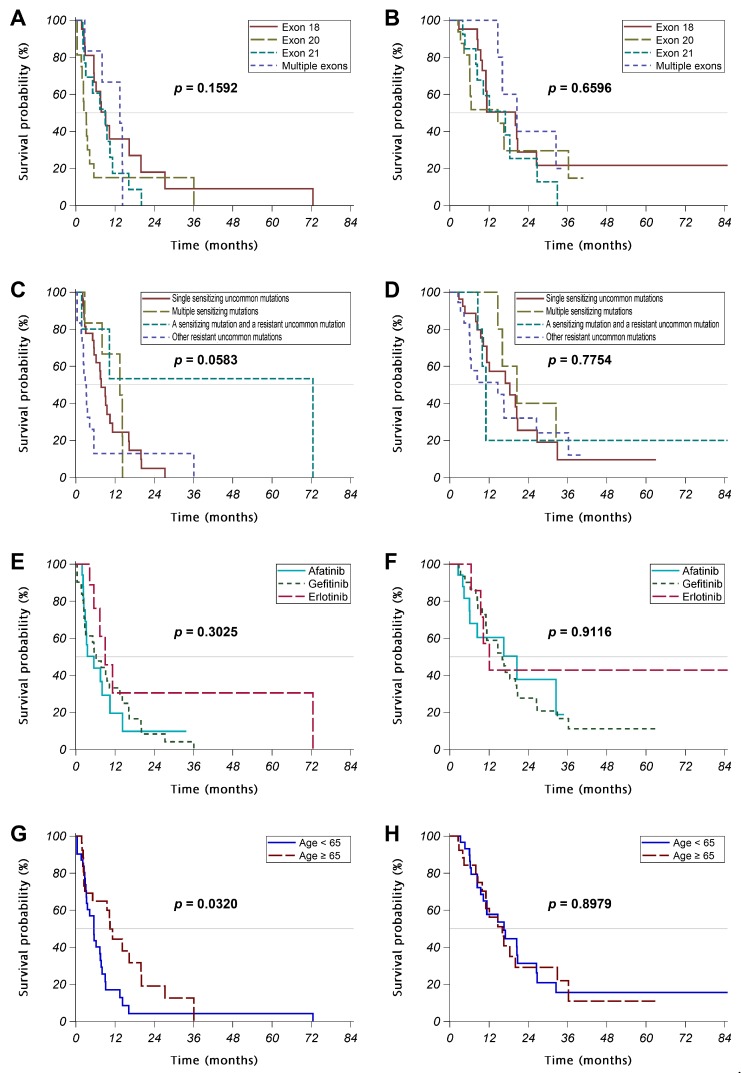
Kaplan-Meier curves of the progression-free survival (PFS) (**A**,**C**,**E**,**G**) and overall survival (OS) (**B**,**D**,**F**,**H**) of the patients receiving a first-line EGFR TKI, while classified the patients by (**A**,**B**) the exon of EGFR mutation, (**C**,**D**) the drug sensitivity of EGFR mutation, (**E**,**F**) the EGFR TKI used, and (**G**,**H**) the age group. Abbreviation: PFS = progression-free survival; OS = overall survival.

In the stage IV patients receiving a first-line *EGFR* TKI, 21 (37%), 16 (28%), 13 (23%), and 7 (12%) had mutation in exons 18, 20, 21, and multiple exons, respectively (Table 1 and Table 2). Patients with their tumors harboring mutations in different exons of *EGFR* were similar in age, sex, smoking history, and performance status. The response rate and disease control rate were significantly higher in those having mutations in multiple exons (Table 2). However, no significant difference was noted in the PFS or OS between groups (Table 2, Figure 1A,B).

While classifying the *EGFR* mutation pattern by drug sensitivity, the patients were reclassified into 27 (47%) “single sensitizing uncommon mutation”, 7 (12%) “multiple sensitizing mutations”, 5 (9%) “a sensitizing mutation and a resistant uncommon mutation” (all of them were exon 18 G719X with another exon 18 point mutation), and 18 (32%) “other resistant uncommon mutations” (Table 1 and Table 3). Patients with their tumors harboring different *EGFR* mutation patterns showed significantly different treatment responses to *EGFR* TKIs, while those with “multiple sensitizing mutations” had the best response rate to TKIs (Table 3). However, no significant difference was noted in the PFS or OS between groups (Table 3, Figure 1C,D).

### 3.3. Different TKIs Showed Similar Treatment Response

In the 57 patients with stage IV disease receiving an *EGFR* TKI as their first-line treatment, 17 (30%), 31 (54%), and 9 (16%) patients used afatinib, gefitinib, and erlotinib, respectively (Table 4). The response rate to the TKI was similar in three groups, while patients receiving erlotinib seemed to have a trend for better disease control rate (*p* = 0.0914). No significant difference was noted in the PFS or OS between patients receiving different first-line *EGFR* TKIs (Table 4, Figure 1E,F).

### 3.4. Elder Patients Had Better PFS but Similar OS

In the patients with stage IV disease receiving first-line *EGFR* TKI, 26 (46%) were elder (age ≥ 65 years old) patients (Table 5). There were significantly more male patients in the elder group than in the younger group (62% vs. 29%, *p* = 0.0176). The elder patients had significantly poorer performance status than the younger patients (percentage of patients with EGOG 2-4: 46% vs. 19%, *p* = 0.0453). The response rate to TKI and disease control rate with TKI was similar between the elder and younger patients. The elder patients had a significantly longer PFS than the younger patients (median PFS: 10.5 vs. 5.5 months, *p* = 0.0320), whereas the OS was similar between groups (*p* = 0.8979) (Table 5, Figure 1G,H).

### 3.5. Factors Related to PFS and OS

Cox regression analyses were used to identify prognostic factors related to the PFS and OS in patients with stage IV lung adenocarcinoma harboring uncommon mutation treated with a first-line *EGFR* TKI. Univariate analyses identified significantly good prognostic factors for PFS included elder age (HR = 0.52 [95% CI: 0.28–0.95], *p* = 0.0349) and having a sensitizing mutation and a resistant uncommon mutation (HR = 0.19 [95% CI: 0.04–0.85], *p* = 0.0296) (Table 6). As for the metastatic site, leptomeningeal metastasis was the only one associated with a trend for poorer PFS (Table A3). On multivariable analyses, including maximal models and reduced models developed with backward variable selection method, elder age and female sex were independent prognostic factors for better PFS, while the mutation patterns also had significant effects on PFS. Smoking history, performance status, or the types of *EGFR* TKIs did not significantly affect the PFS.

Female sex and never smoker were significant factors for better OS on univariate analyses but became insignificant in the multivariable models (Table 7). As for metastatic site, brain and leptomeningeal metastases were associated with poorer OS (Table A3), while this finding might be biased by small sample size. Better initial performance status remained a significant factor for better OS in the univariate model and multivariable models, including maximal models and the reduced model. The mutation patterns or the types of *EGFR* TKIs did not significantly affect the OS. Elder age was not associated with a better OS on univariate analysis, whereas multivariable models showed that elder age had a trend for better OS.

## 4. Discussion

In the past decade, *EGFR* TKIs had replaced platinum-based chemotherapy to be a standard therapy in patients of NSCLCs harboring *EGFR* mutation, because many phase 3 randomized controlled trials demonstrated that patients receiving *EGFR* TKIs had better response rate and longer PFS. However, most of these studies enrolled common mutations, including exon 19 deletion mutation and exon 21 L858R point mutation. To date, the effect of *EGFR* TKIs in NSCLCs with uncommon mutations are not completely understood. This study is probably one of the largest-scale comprehensive studies about the effect of a first-line *EGFR* TKI in patients having lung adenocarcinoma harboring uncommon *EGFR* mutations. We found that the elder patients, although having significantly poorer initial performance status, had a significantly longer PFS than the younger patients.

Approximately 90% *EGFR* mutations are exon 19 deletion or exon 21 L858R point mutation, and both of them are sensitizing to *EGFR* TKIs. However, uncommon mutations, also known as non-classic or rare mutations, have been rarely discussed, because they are rarely seen and only parts of them receive first- or second-generation *EGFR* TKIs. Uncommon or non-classical mutation is a heterogeneous group of molecular alterations with variable responses to *EGFR*-targeted drugs. Only several retrospective small-scale studies to date tried to identify the clinical efficacy in these patients but the results were inconclusive [8,9,10,13,14,22,23,24]. Exon 18 G719X, exon 20 S768I, and exon 21 L861Q are generally considered as sensitizing uncommon mutations. In contrast, the de novo exon 20 T790M mutation and exon 20 insertion predict primary resistance to clinically achievable levels of *EGFR* TKIs [8,9,14].

Patients with lung cancer harboring a “single sensitizing uncommon mutation”, including G719X, S768I, and L861Q, usually had a good treatment response to EGFR TKI, although the response was generally still inferior to those harboring common sensitizing mutations. Chen et al. reported that the patients with NSCLC harboring a single uncommon sensitizing mutation (either G719X, L861Q, or S768I) had a response rate of 32.4% and disease control rate of 83.8% [14]. Yun et al. showed G719S mutation had a 14-fold higher affinity to adenosine triphosphate than the wild-type but a weaker affinity to gefitinib than L858R mutation, suggesting that gefitinib could inhibit cancers harboring G719S mutation with less effectiveness [6]. Both studies by Chen et al. [7] and Kancha et al. [25] indicated S768I mutation had resistance to *EGFR* TKI in vitro, but some case reports indicated S768I mutation was still responsive to *EGFR* TKI clinically. In our current study, the patients with adenocarcinoma harboring a single sensitizing uncommon mutation had a fine initial treatment response to first-line *EGFR* TKIs (disease control rate of 85%), PFS (median, 7.7 months), and OS (median, 18.4 months).

Some uncommon mutations are sporadic, and most of them are resistant to an *EGFR* TKI. Shen et al. showed that G779F, L747P, and M825L with R831C were sensitizing mutations, whereas V717G, I715V, K716E, and complex non-classic mutation, such as V742F, A743V, and H773R, were resistant mutations [9]. Ibrahim et al. reported a patient with stage IV lung adenocarcinoma harboring delE709 T710insD in exon 18 who had a good response to afatinib [26].

In patients with lung cancer harboring uncommon mutation, a few of them had compound mutations. “Multiple sensitizing mutations” is a complex group, mostly having co-existing sensitizing mutations in different exons. Chiu et al. reported that co-existence of G719X and L861Q had a high objective response rate of 88.9% but co-existence of G719X and S768I had an objective response rate of only 50%. They further indicated that patients with compound uncommon *EGFR* mutations (G719X + L861Q or G719X + S768I) had a significantly longer PFS than the patients with a single mutation did (median PFS: 11.9 vs. 6.5 months; *p* = 0.010) [13]. In a report by Kobayashi et al., two cases with tumor harboring G719X + S768I had partial responses to erlotinib but the PFSs were only 5 and 7 months [11]. Tu et al. reported that patients with lung cancer harboring compound L858R mutations and G719X mutations, which comprised the majority of uncommon *EGFR* mutations, had objective response rates of 75% and 50% and median PFSs of 15.2 and 11.6 months, respectively [8]. However, Chen et al. presented several cases with cancer harboring G719X + L861Q or G719X + S768I, who had no response to *EGFR* TKI [14]. Our present study indicated that the “multiple sensitizing mutations” group had a significantly better initial treatment response to first-line *EGFR* TKI (response rate of 71% and disease control rate of 86%), and a fine PFS (median, 13.5 months), and OS (median, 20.5 months). Nevertheless, these cases were very rare and heterogeneous, and a simple conclusion is hard to be made.

As another type of compound mutation, an even smaller group of patients had their cancer harboring “a sensitizing (common or uncommon) mutation and a resistant uncommon mutation.” In the study by Peng et al., five patients had adenocarcinoma harboring L858R compound mutations, including one case of L858R + S768I. The four cases with L858R and a resistant uncommon mutation had a stable disease as the initial treatment response to gefitinib treatment and had a median PFS of 10 months [10]. In the report by Kobayashi et al., a case with adenocarcinoma harboring G719X and a resistant uncommon mutation and two cases with adenocarcinoma harboring L858R and a resistant uncommon mutation had a partial response as the initial treatment response to first-line erlotinib treatment [11]. In another report, a patient with co-existence of I706T and G719A had a good response and a PFS of at least 22 months, but a patient with co-existence of E709K and G719A had a very poor response to *EGFR* TKI [27]. Kauffmann-Guerrero et al. reported a patient with co-existence of G857E and R836R in exon 21, who had a very poor response to *EGFR* TKI [12]. In our current study, interestingly, all of five patients with stage IV adenocarcinoma harboring a sensitizing mutation and a resistant uncommon mutation involved two point mutations in a single exon (exon 18 G719X with another exon 18 point mutation). These patients, treated with a first-line *EGFR* TKI, had a fine initial treatment response rate (40%), disease control rate (80%) and a very long PFS (median PFS: 72.5 months).

Patients with lung cancer harboring a “single sensitizing uncommon mutation”, including G719X, S768I, and L861Q, usually had a good treatment response to EGFR TKI, although the response was generally still inferior to those harboring common sensitizing mutations as the report of Chiu et al. [13]. 

Traditionally, *EGFR* TKIs are less effective to uncommon mutations than to common sensitizing mutations. Some studies indicated that the irreversible *EGFR* TKI such as afatinib might be more effective for the patients with lung cancer harboring an uncommon mutation [9,15]. Yang et al. summarized Lux-Lung series and indicated afatinib was active in NSCLC harboring certain types of uncommon *EGFR* mutations, especially G719X, S768I, and L861Q but was less active in those harboring other uncommon mutation types [15]. In the study by Shen et al., the analysis of 51 patients having lung adenocarcinoma harboring uncommon *EGFR* mutations except for exon 20 insertion showed that afatinib provided significantly longer PFS than gefitinib/erlotinib did (median PFS: 11.0 vs. 3.6 months, *p* = 0.03) [9]. However, the inclusion of patients with stage IIIB disease and those receiving an *EGFR* TKI as the second or third line of treatment, as well as the exclusion of five cases with exon 20 insertion, might confound the analysis. Our current study clearly focused on a clear group with patients receiving an *EGFR* TKI as the first-line treatment for their stage IV lung adenocarcinoma harboring uncommon mutations and found no significant difference in the initial treatment response, PFS, and OS between patients receiving different *EGFR* TKIs.

Several studies have tried to demonstrate the prognostic factors of lung cancer patients. In a study of patients receiving *EGFR* TKI (as any line of treatment) for advanced lung adenocarcinoma harboring (either common or uncommon) *EGFR* mutation by Chiu et al. [13], female sex, elderly (age > 70 years), and common *EGFR* mutation were independent factors suggesting better PFS. However, the analysis might be confounded by the enrollment of patients with stage IIIB disease and those receiving *EGFR* TKI as the second- or later-line treatment and by the absence of patients receiving afatinib. In addition, these studies did not report the prognostic factors specifically in the uncommon mutation group. In the current study, we first identified that the elder was an independent prognostic factor for better PFS in the patients treated with a first-line *EGFR* TKI for their stage IV lung adenocarcinoma harboring uncommon mutation. 

Furthermore, large-scale prospective studies are warranted to elucidate the efficacy and prognostic factors of different EGFR TKIs in patients with different rare or compound mutations. Osimertinib, a 3rd generation EGFR TKI, has been used to treat the de novo T790M mutation [28], and several case reports have demonstrated that it may be effective in some patients harboring uncommon mutations [29,30]. However, more evidence is still needed.

Several limitations of this study should be addressed. First of all, this study was retrospective, and selection bias cannot be completely avoided. Many patients with uncommon mutation did not receive *EGFR* TKI because previous studies had shown poor clinical efficacy of *EGFR* TKIs in this group of patients. Second, some patients may not have undergone follow-up imaging on schedule. Although regular imaging follow-up is required for the patients to receive reimbursements for *EGFR* TKIs every three months, missing a follow-up exam still occurs. This and the unavoidable bias from a retrospective study design may be overcome through future well-designed prospective studies. Third, due to the uncommon entity, we might be unable to collect sufficient cases to do the analysis. However, this study might be one of the largest-scale comprehensive studies about this topic to date.

## 5. Conclusions

In conclusion, the elder patients with stage IV lung adenocarcinoma harboring uncommon *EGFR* mutation, although having significantly poor performance status, may have a longer PFS than the younger patients, while treated with a first-line *EGFR* TKI, but the OS was similar in the elder and younger patients. No significant difference in PFS or OS was observed in patients receiving either gefitinib, erlotinib, or afatinib as the first-line treatment. A further large-scale study is necessary to validate our findings, as well as to determine the best treatment modality for patients with lung adenocarcinoma harboring uncommon *EGFR* mutation.

## Figures and Tables

**Table 1:** Characteristics of the patients with stage IV lung cancer harboring uncommon mutation.

	All Stage IV Patients	Patients Receiving First-Line TKI
n	62	57
Age (year), mean ± SD	65 ± 12	66 ± 12
Age (year), median (IQR)	64 (56–74)	65 (56–75)
Sex, n (%)		
Female	36 (58%)	32 (56%)
Male	26 (42%)	25 (44%)
Smoking history, n (%)		
Never	44 (71%)	40 (70%)
Current	18 (29%)	17 (30%)
Performance status, n (%)		
ECOG 0–1	44 (71%)	39 (68%)
ECOG 2–4	18 (29%)	18 (32%)
TKI medication, n (%)		
Afatinib	19 (31%)	17 (30%)
Gefitinib	32 (52%)	31 (54%)
Erlotinib	11 (18%)	9 (16%)
TKI use, n (%)		
1st-line treatment	57 (92%)	57 (100%)
2nd-line treatment	3 (5%)	
after 2nd-line treatment	2 (3%)	
Number of metastatic sites, n (%)		
=1	29 (47%)	27 (47%)
≥2	33 (53%)	30 (53%)
Metastatic site, n (%)		
Brain	16 (26%)	14 (25%)
Leptomeningeal	2 (3%)	2 (4%)
Lung	23 (37%)	20 (35%)
Bone	32 (52%)	30 (53%)
Pleural	29 (47%)	29 (51%)
Pericardial	5 (8%)	5 (9%)
Liver	10 (16%)	9 (16%)
Adrenal	5 (8%)	5 (9%)
Renal	1 (2%)	0 (0%)
Mutation site, n (%)		
Exon 18 G719X	14 (23%)	14 (25%)
Exon 18 other mutation	2 (3%)	2 (4%)
Exon 18 G719X + exon 18 other mutation	5 (8%)	5 (9%)
Exon 20 insertion	16 (26%)	13 (23%)
Exon 20 point mutation	3 (5%)	3 (5%)
Exon 21 L861Q	13 (21%)	13 (23%)
Exon 18 G719X + exon 20 S768I	3 (5%)	3 (5%)
Exon 18 G719X + exon 21 L861Q	2 (3%)	2 (4%)
Exon 21 L858R + exon 20 S768I	3 (5%)	2 (4%)
Exon 21 L858R + exon 20 Q812*	1 (2%)	0 (0%)
Mutation site classified by exon, n (%)		
Mutation only in exon 18	21 (34%)	21 (37%)
Mutation only in exon 20	19 (31%)	16 (28%)
Mutation only in exon 21	13 (21%)	13 (23%)
Mutations in multiple exons	9 (15%)	7 (12%)
Mutation site classified by susceptibility ^†^, n (%)		
Single sensitizing uncommon mutation	27 (44%)	27 (47%)
Multiple sensitizing mutations	8 (13%)	7 (12%)
A sensitizing mutation and a resistant uncommon mutation	6 (10%)	5 (9%)
Other resistant uncommon mutations	21 (34%)	18 (32%)
Initial response to TKI, n (%)		
Partial response	21 (34%)	19 (33%)
Stable disease	24 (39%)	22 (39%)
Progressive disease	17 (27%)	16 (28%)
Response rate to TKI	21 (34%)	19 (33%)
Disease control rate to TKI	45 (73%)	41 (72%)
Median follow-up time (months)	13.0	11.5
Median PFS on TKI (months)	7.5	7.4
Median OS (month)	17.0	16.1

Abbreviations: ECOG = Eastern Cooperative Oncology Group; TTF-1 = thyroid transcription factor 1. ^†^ Mutation site classified by susceptibility: “Single sensitizing uncommon mutation” included exon18 G719X and exon21 L861Q; “multiple sensitizing mutations” included exon18 G719X + exon20 S768I, exon18 G719X + exon21 L861Q, and exon21 L858R + exon20 S768I; “a sensitizing mutation and a resistant uncommon mutation” included exon18 G719X + exon18 other and exon21 L858R + exon20 Q812*; “other resistant uncommon mutations” included exon 18 other mutation, exon 20 insertion, and exon 20 point mutation.

**Table 2:** Characteristics of the patients with stage IV lung cancer harboring uncommon mutation receiving a first-line tyrosine kinase inhibitor (TKI), classified by the exon of epidermal growth factor receptor (*EGFR*) mutation.

	Mutation Only in exon 18	Mutation Only in exon 20	Mutation Only in exon 21	Mutations in Multiple Exons	*p* Value *
n	21 (37%)	16 (28%)	13 (23%)	7 (12%)	
Age (year), mean ± SD	66 ± 13	63 ± 10	69 ± 13	64 ± 11	0.6964
Age (year), median (IQR)	64 (55–79)	65 (55–70)	67 (56–78)	63 (60–73)	0.6964
Age (year), n (%)					0.7749
<65	11 (52%)	9 (56%)	6 (46%)	5 (71%)	
≥65	10 (48%)	7 (44%)	7 (54%)	2 (29%)	
Sex, n (%)					1.0000
Female	12 (57%)	9 (56%)	7 (54%)	4 (57%)	
Male	9 (43%)	7 (44%)	6 (46%)	3 (43%)	
Smoking history, n (%)					0.3188
Never	14 (67%)	11 (69%)	8 (62%)	7 (100%)	
Current	7 (33%)	5 (31%)	5 (38%)	0 (0%)	
Performance status, n (%)					0.0799
ECOG 0–1	17 (81%)	7 (44%)	9 (69%)	6 (86%)	
ECOG 2–4	4 (19%)	9 (56%)	4 (31%)	1 (14%)	
No. of metastatic sites, n (%)					0.3715
=1	11 (52%)	7 (44%)	4 (31%)	5 (71%)	
≥2	10 (48%)	9 (56%)	9 (69%)	2 (29%)	
Metastatic site, n (%)					
Brain	5 (24%)	5 (31%)	3 (23%)	1 (14%)	0.8976
Leptomeningeal	0 (0%)	2 (13%)	0 (0%)	0 (0%)	0.2644
Lung	4 (19%)	5 (31%)	9 (69%)	2 (29%)	0.0288
Bone	11 (52%)	8 (50%)	9 (69%)	2 (29%)	0.3946
Pleural	11 (52%)	8 (50%)	7 (54%)	3 (43%)	1.0000
Pericardial	3 (14%)	0 (0%)	1 (8%)	1 (14%)	0.4222
Liver	1 (5%)	2 (13%)	6 (46%)	0 (0%)	0.0109
Adrenal	3 (14%)	2 (13%)	0 (0%)	0 (0%)	0.5324
TKI medication, n (%)					0.2374
Afatinib	4 (19%)	8 (50%)	2 (15%)	3 (43%)	
Gefitinib	14 (67%)	7 (44%)	7 (54%)	3 (43%)	
Erlotinib	3 (14%)	1 (6%)	4 (31%)	1 (14%)	
Initial response to TKI, n (%)					0.0212
Partial response	8 (38%)	1 (6%)	5 (38%)	5 (71%)	
Stable disease	10 (48%)	6 (38%)	5 (38%)	1 (14%)	
Progressive disease	3 (14%)	9 (56%)	3 (23%)	1 (14%)	
Response rate to TKI	8 (38%)	1 (6%)	5 (38%)	5 (71%)	0.0118
Disease control rate with TKI	18 (86%)	7 (44%)	10 (77%)	6 (86%)	0.0357
Median PFS on TKI (month)	9.2	2.8	9.0	13.5	0.1592
Median OS (month)	20.0	14.7	17.0	20.5	0.6596

* Kruskal-Wallis test or Fisher’s exact test or Log-rank test.

**Table 3:** Characteristics of the patients with stage IV lung cancer harboring uncommon mutation receiving a first-line tyrosine kinase inhibitor (TKI), classified by the drug susceptibility of EGFR mutation ^†^.

	Single Sensitizing Uncommon Mutation	Multiple Sensitizing Uncommon Mutations	A Sensitizing Mutation and a Resistant Uncommon Mutation	Other Resistant Uncommon Mutations	*p* Value *
n	27 (47%)	7 (12%)	5 (9%)	18 (32%)	
Age (year), mean ± SD	67 ± 12	64 ± 11	77 ± 13	61 ± 10	0.0971
Age (year), median (IQR)	64 (56–78)	63 (60–73)	84 (75–85)	64 (49–70)	0.0971
Age (year), n (%)					0.3426
<65	14 (52%)	5 (71%)	1 (20%)	11 (61%)	
≥65	13 (48%)	2 (29%)	4 (80%)	7 (39%)	
Sex, n (%)					0.4195
Female	17 (63%)	4 (57%)	1 (20%)	10 (56%)	
Male	10 (37%)	3 (43%)	4 (80%)	8 (44%)	
Smoking history, n (%)					0.0286
Never	20 (74%)	7 (100%)	1 (20%)	12 (67%)	
Current	7 (26%)	0 (0%)	4 (80%)	6 (33%)	
Performance status, n (%)					0.1784
ECOG 0-1	21 (78%)	6 (86%)	3 (60%)	9 (50%)	
ECOG 2-4	6 (22%)	1 (14%)	2 (40%)	9 (50%)	
Number of metastatic sites, n (%)					0.1168
=1	9 (33%)	5 (71%)	4 (80%)	9 (50%)	
≥2	18 (67%)	2 (29%)	1 (20%)	9 (50%)	
Metastatic site, n (%)					
Brain	7 (26%)	1 (14%)	1 (20%)	5 (28%)	0.9629
Leptomeningeal	0 (0%)	0 (0%)	0 (0%)	2 (11%)	0.3571
Lung	13 (48%)	2 (29%)	0 (0%)	5 (28%)	0.1805
Bone	18 (67%)	2 (29%)	1 (20%)	9 (50%)	0.1168
Pleural	15 (56%)	3 (43%)	3 (60%)	8 (44%)	0.8305
Pericardial	3 (11%)	1 (14%)	0 (0%)	1 (6%)	0.8033
Liver	7 (26%)	0 (0%)	0 (0%)	2 (11%)	0.3156
Adrenal	2 (7%)	0 (0%)	1 (20%)	2 (11%)	0.5715
TKI medication, n (%)					0.1599
Afatinib	5 (19%)	3 (43%)	0 (0%)	9 (50%)	
Gefitinib	16 (59%)	3 (43%)	4 (80%)	8 (44%)	
Erlotinib	6 (22%)	1 (14%)	1 (20%)	1 (6%)	
Initial response to TKI, n (%)					0.0062
Partial response	11 (41%)	5 (71%)	2 (40%)	1 (6%)	
Stable disease	12 (44%)	1 (14%)	2 (40%)	7 (39%)	
Progressive disease	4 (15%)	1 (14%)	1 (20%)	10 (56%)	
Response rate to TKI	11 (41%)	5 (71%)	2 (40%)	1 (6%)	0.0037
Disease control rate with TKI	23 (85%)	6 (86%)	4 (80%)	8 (44%)	0.0188
Median PFS on TKI (month)	7.7	13.5	72.5	3.1	0.0583
Median OS (month)	18.4	20.5	11.0	14.7	0.7754

* Kruskal-Wallis test or Fisher’s exact test or Log-rank test. ^†^ Mutation site classified by susceptibility: “Single sensitizing uncommon mutation” included exon18 G719X and exon21 L861Q; “multiple sensitizing mutations” included exon18 G719X + exon20 S768I, exon18 G719X + exon21 L861Q, and exon21 L858R + exon20 S768I; “a sensitizing mutation and a resistant uncommon mutation” included exon18 G719X + exon18 other and exon21 L858R + exon20 Q812*; “other resistant uncommon mutations” included exon 18 other mutation, exon 20 insertion, and exon 20 point mutation.

**Table 4:** Characteristics of the patients with stage IV lung cancer harboring uncommon mutation receiving a first-line tyrosine kinase inhibitor (TKI), classified by the drug used.

	Afatinib	Gefitinib	Erlotinib	*p* Value *
n	17 (30%)	31 (54%)	9 (16%)	
Age (year), mean ± SD	64 ± 9	67 ± 14	62 ± 11	0.4356
Age (year), median (IQR)	65 (61–70)	67 (56–80)	61 (55–67)	0.4356
Age (year), n (%)				0.5820
<65	11 (65%)	15 (48%)	5 (56%)	
≥65	6 (35%)	16 (52%)	4 (44%)	
Sex, n (%)				0.1084
Female	8 (47%)	16 (52%)	8 (89%)	0.2477
Male	9 (53%)	15 (48%)	1 (11%)	
Smoking history, n (%)				0.2477
Never	13 (76%)	19 (61%)	8 (89%)	
Current	4 (24%)	12 (39%)	1 (11%)	
Performance status, n (%)				0.3383
ECOG 0–1	10 (59%)	21 (68%)	8 (89%)	
ECOG 2–4	7 (41%)	10 (32%)	1 (11%)	
Number of metastatic sites, n (%)				0.3891
=1	6 (35%)	17 (55%)	4 (44%)	
≥2	11 (65%)	14 (45%)	5 (56%)	
Metastatic site, n (%)				
Brain	6 (35%)	5 (16%)	3 (33%)	0.2500
Leptomeningeal	2 (12%)	0 (0%)	0 (0%)	0.1078
Lung	9 (53%)	8 (26%)	3 (33%)	0.1661
Bone	8 (47%)	17 (55%)	5 (56%)	0.9344
Pleural	7 (41%)	19 (61%)	3 (33%)	0.2398
Pericardial	4 (24%)	1 (3%)	0 (0%)	0.0542
Liver	3 (18%)	5 (16%)	1 (11%)	1.0000
Adrenal	2 (12%)	2 (6%)	1 (11%)	0.6897
Mutation site classified by exon, n (%)				0.2374
Mutation only in exon 18	4 (24%)	14 (45%)	3 (33%)	
Mutation only in exon 20	8 (47%)	7 (23%)	1 (11%)	
Mutation only in exon 21	2 (12%)	7 (23%)	4 (44%)	
Mutations in multiple exons	3 (18%)	3 (10%)	1 (11%)	
Mutation site classified by susceptibility †, n (%)				0.1599
Single sensitizing uncommon mutation	5 (29%)	16 (52%)	6 (67%)	
Multiple sensitizing mutations	3 (18%)	3 (10%)	1 (11%)	
A sensitizing mutation and a resistant uncommon mutation	0 (0%)	4 (13%)	1 (11%)	
Other resistant uncommon mutations	9 (53%)	8 (26%)	1 (11%)	
Initial response to TKI, n (%)				0.1943
Partial response	7 (41%)	9 (29%)	3 (33%)	
Stable disease	5 (29%)	11 (35%)	6 (67%)	
Progressive disease	5 (29%)	11 (35%)	0 (0%)	
Response rate to TKI	7 (41%)	9 (29%)	3 (33%)	0.7450
Disease control rate with TKI	12 (71%)	20 (65%)	9 (100%)	0.0914
Median PFS on TKI (month)	5.5	6.2	9.0	0.3025
Median OS (month)	20.5	16.1	12.1	0.9116

* Kruskal-Wallis test or Fisher’s exact test or Log-rank test. ^†^ Mutation site classified by susceptibility: “Single sensitizing uncommon mutation” included exon18 G719X and exon21 L861Q; “multiple sensitizing mutations” included exon18 G719X + exon20 S768I, exon18 G719X + exon21 L861Q, and exon21 L858R + exon20 S768I; “a sensitizing mutation and a resistant uncommon mutation” included exon18 G719X + exon18 other and exon21 L858R + exon20 Q812*; “other resistant uncommon mutations” included exon 18 other mutation, exon 20 insertion, and exon 20 point mutation.

**Table 5:** Characteristics of the patients with stage IV lung cancer harboring uncommon mutation receiving a first-line tyrosine kinase inhibitor (TKI), classified by the age group.

	Age < 65	Age ≥ 65	*p* Value *
n	31 (54%)	26 (46%)	
Age (year), mean ± SD	57 ± 7	76 ± 7	<0.0001
Age (year), median (IQR)	59 (50–62)	77 (71–81)	<0.0001
Sex, n (%)			0.0176
Female	22 (71%)	10 (38%)	
Male	9 (29%)	16 (62%)	
Smoking history, n (%)			0.2494
Never	24 (77%)	16 (62%)	
Current	7 (23%)	10 (38%)	
Performance status, n (%)			0.0453
ECOG 0–1	25 (81%)	14 (54%)	
ECOG 2–4	6 (19%)	12 (46%)	
Number of metastatic sites, n (%)			0.4311
=1	13 (42%)	14 (54%)	
≥2	18 (58%)	12 (46%)	
Metastatic site, n (%)			
Brain	9 (29%)	5 (19%)	0.5392
Leptomeningeal	1 (3%)	1 (4%)	1.0000
Lung	11 (35%)	9 (35%)	1.0000
Bone	17 (55%)	13 (50%)	0.7931
Pleural	14 (45%)	15 (58%)	0.4287
Pericardial	4 (13%)	1 (4%)	0.3624
Liver	6 (19%)	3 (12%)	0.4876
Adrenal	3 (10%)	2 (8%)	1.0000
Mutation site classified by exon, n (%)			0.7749
Mutation only in exon 18	11 (35%)	10 (38%)	
Mutation only in exon 20	9 (29%)	7 (27%)	
Mutation only in exon 21	6 (19%)	7 (27%)	
Mutations in multiple exons	5 (16%)	2 (8%)	
Mutation site classified by susceptibility ^†^, n (%)			0.3426
Single sensitizing uncommon mutation	14 (45%)	13 (50%)	
Multiple sensitizing mutations	5 (16%)	2 (8%)	
A sensitizing mutation and a resistant uncommon mutation	1 (3%)	4 (15%)	
Other resistant uncommon mutations	11 (35%)	7 (27%)	
TKI medication, n (%)			0.5820
Afatinib	11 (35%)	6 (23%)	
Gefitinib	15 (48%)	16 (62%)	
Erlotinib	5 (16%)	4 (15%)	
Initial response to TKI, n (%)			0.7086
Partial response	9 (29%)	10 (38%)	
Stable disease	12 (39%)	10 (38%)	
Progressive disease	10 (32%)	6 (23%)	
Response rate to TKI	9 (29%)	10 (38%)	0.5748
Disease control rate with TKI	21 (68%)	20 (77%)	0.5581
Median PFS on TKI (month)	5.5	10.5	0.0320
Median OS (month)	16.6	16.1	0.8979

* Kruskal-Wallis test or Fisher’s exact test or Log-rank test. ^†^ Mutation site classified by susceptibility: “Single sensitizing uncommon mutation” included exon18 G719X and exon21 L861Q; “multiple sensitizing mutations” included exon18 G719X + exon20 S768I, exon18 G719X + exon21 L861Q, and exon21 L858R + exon20 S768I; “a sensitizing mutation and a resistant uncommon mutation” included exon18 G719X + exon18 other and exon21 L858R + exon20 Q812*; “other resistant uncommon mutations” included exon 18 other mutation, exon 20 insertion, and exon 20 point mutation.

**Table 6:** Cox proportional hazards regression analyses for identifying the relationship between clinical features and progression-free survival in patients with stage IV lung cancer harboring uncommon mutation receiving first-line TKI.

	Univariate Analysis	Multivariable Analyses			
		Model 1	Model 2	Model 3	Model 4
Sex					
Female	1.00	1.00	1.00	1.00	1.00
Male	1.50 [0.83–2.72]	2.83 [1.04–7.72]	2.90 [1.07–7.88]	2.13 [1.11–4.09]	2.10 [1.11–3.97]
Age (year)					
<65	1.00	1.00	1.00	1.00	1.00
≥65	0.52 [0.28–0.95]	0.27 [0.12–0.58]	0.28 [0.13–0.60]	0.35 [0.17–0.72]	0.35 [0.17–0.73]
Smoking history					
Never	1.00	1.00	1.00		
Current	1.33 [0.70–2.52]	0.44 [0.14–1.42]	0.46 [0.15–1.46]		
Performance status					
ECOG 0–1	1.00	1.00	1.00		
ECOG 2–4	1.60 [0.85–3.02]	1.51 [0.60–3.84]	1.52 [0.62–3.74]		
Number of metastatic sites					
=1	1.00	1.00	1.00	1.00	1.00
≥2	1.60 [0.85–3.02]	1.65 [0.78–3.51]	1.87 [0.89–3.94]	1.75 [0.87–3.54]	1.73 [0.84–3.58]
Mutation site classified by exon					
Mutation only in exon 18	1.02 [0.36–2.84]	1.31 [0.42–4.09]		1.11 [0.38–3.20]	
Mutation only in exon 20	2.25 [0.78–6.47]	3.70 [0.93–14.83]		2.84 [0.94–8.60]	
Mutation only in exon 21	1.53 [0.54–4.38]	1.84 [0.53–6.43]		1.59 [0.51–5.01]	
Mutations in multiple exons	1.00	1.00		1.00	
Mutation site classified by susceptibility ^†^					
Single sensitizing uncommon mutation	0.57 [0.29–1.10]		0.39 [0.16–0.96]		0.48 [0.23–1.02]
Multiple sensitizing mutations	0.43 [0.15–1.21]		0.27 [0.07–1.01]		0.34 [0.12–1.01]
A sensitizing mutation and a resistant uncommon mutation	0.19 [0.04–0.85]		0.20 [0.04–1.08]		0.18 [0.04–0.83]
Other resistant uncommon mutations	1.00		1.00		1.00
TKI medication					
Afatinib	1.00	1.00	1.00		
Gefitinib	0.90 [0.46–1.76]	1.85 [0.76–4.46]	1.90 [0.82–4.43]		
Erlotinib	0.46 [0.16–1.30]	0.91 [0.27–3.05]	1.24 [0.37–4.19]		

Model 1 and Model 2 are maximal models. Model 3 and Model 4 are reduced multivariable models developed with backward variable selection method, keeping only variables with *p* value less than 0.15, from Model 1 and Model 2, respectively. ^†^ Mutation site classified by susceptibility: “Single sensitizing uncommon mutation” included exon18 G719X and exon21 L861Q; “multiple sensitizing mutations” included exon18 G719X + exon20 S768I, exon18 G719X + exon21 L861Q, and exon21 L858R + exon20 S768I; “a sensitizing mutation and a resistant uncommon mutation” included exon18 G719X + exon18 other and exon21 L858R + exon20 Q812*; “other resistant uncommon mutations” included exon 18 other mutation, exon 20 insertion, and exon 20 point mutation.

**Table 7:** Cox proportional hazards regression analyses for identifying the relationship between clinical features and overall survival in patients with stage IV lung cancer harboring uncommon mutation receiving first-line TKI.

	Univariate Analysis	Multivariable Analyses		
		Model 1	Model 2	Model 3
Sex				
Female	1.00	1.00	1.00	1.00
Male	2.31 [1.19–4.47]	1.81 [0.65–4.98]	1.86 [0.67–5.16]	1.83 [0.88–3.78]
Age (year)				
<65	1.00	1.00	1.00	1.00
≥65	1.04 [0.55–1.98]	0.45 [0.18–1.12]	0.46 [0.19–1.15]	0.47 [0.20–1.14]
Smoking history				
Never	1.00	1.00	1.00	
Current	2.40 [1.21–4.74]	1.11 [0.37–3.3]	1.1 [0.36–3.33]	
Performance status				
ECOG 0–1	1.00	1.00	1.00	1.00
ECOG 2–4	2.77 [1.42–5.39]	3.43 [1.21–9.75]	3.32 [1.19–9.28]	3.40 [1.32–8.72]
Number of metastatic sites				
=1	1.00	1.00	1.00	
≥2	2.77 [1.42–5.39]	1.10 [0.49–2.46]	1.20 [0.55–2.60]	
Mutation site classified by exon				
Mutation only in exon 18	1.39 [0.45–4.30]	1.56 [0.43–5.62]		
Mutation only in exon 20	1.73 [0.55–5.48]	1.26 [0.34–4.73]		
Mutation only in exon 21	1.95 [0.61–6.24]	1.90 [0.43–8.48]		
Mutations in multiple exons	1.00	1.00		
Mutation site classified by susceptibility				
Single sensitizing uncommon mutation	0.86 [0.41–1.78]		1.15 [0.49–2.7]	
Multiple sensitizing mutations	0.56 [0.18–1.74]		0.75 [0.21–2.67]	
A sensitizing mutation and a resistant uncommon mutation	1.01 [0.33–3.12]		1.23 [0.34–4.44]	
Other resistant uncommon mutations	1.00		1.00	
TKI medication				
Afatinib	1.00	1.00	1.00	
Gefitinib	1.04 [0.48–2.24]	0.85 [0.33–2.21]	0.91 [0.35–2.36]	
Erlotinib	0.83 [0.25–2.71]	0.93 [0.22–3.95]	1.01 [0.22–4.49]	

* Model 1 and Model 2 are maximal models. Model 3 is a reduced multivariable models developed with backward variable selection method, keeping only variables with *p* value less than 0.15, from both Model 1 and Model 2. ^†^ Mutation site classified by susceptibility: “Single sensitizing uncommon mutation” included exon18 G719X and exon21 L861Q; “multiple sensitizing mutations” included exon18 G719X + exon20 S768I, exon18 G719X + exon21 L861Q, and exon21 L858R + exon20 S768I; “a sensitizing mutation and a resistant uncommon mutation” included exon18 G719X + exon18 other and exon21 L858R + exon20 Q812*; “other resistant uncommon mutations” included exon 18 other mutation, exon 20 insertion, and exon 20 point mutation.

## Appendix A

**Table A1:** Studies about lung cancer harboring rare EGFR mutations.

Author, Year	N ^†^	EGFR TKIs	Categories of Mutation Types	Response Rate (%)	Median PFS (months)	Median OS (months)
Kobayasi, et al., 2013	8	Erlotinib	Compound mutations (sensitive mutation with other mutation):			
			G719X with others (n = 3)	100%		
			L858R with others (n = 3)	66.7%		
			L861Q + E709A (n = 1)	100%		
			delL747_T751+R776S (n = 1)	100%		
Chiu, et al., 2015	151	Erlotinib, Gefitinib	Uncommon mutation (G719X, L861Q, S768I, G719X + L861Q, and G719X + S768I)	41.6%	7.7	24.0
			Common mutation	66.5%	11.4	29.7
Peng, et al., 2015	6	Gefitinib	L858R compound mutations	16.7%	12.2	28.6
Yang, et al., 2015	75	Afatinib	Group 1: point mutations and duplications in exons 18-21(L861G, G719X, S768I, and other point mutations alone or in combination with each other)	71.1%	10.7	19.4
			Group 2: de-novo T790M mutation in exon 20 (alone or in combination with other mutations)	14.3%	2.9	14.9
			Group 3: exon 20 insertions	8.7%	2.7	9.2
Chen, et al., 2017	66	Icotinib, Gefitinib, Erlotinib, Afatinib	Group 1: sensitizing rare mutations (G719X, L861Q, S768I)	32.4%	4.1	
			Group 2: resistant mutation (exon 20 insertion)	11.1%	3.1	
			Group 3: complex mutation (L858R + T790M)	30%	8.6	
Kauffmann-Guerrero, et al., 2017	12	Erlotinib, Afatinib	Rare mutation	28.5		
			Complex mutation	20%		
Tu, et al., 2018	62	EGFR TKIs	G719X	50%	11.6	25.2
			Exon 20 insertion	0%	3	12.5
			T790M	0%	1	2.4
			Complex L858R	75%	15.2	27.7
			Others	10%	2.9	11.7
Shen, et al., 2018	56	Gefitinib, Erlotinib, Afatinib	Group 1: exon 20 insertions (except A763_Y764 insFQEA)	20%		
			Group 2: non-classical mutations with Del19 or L858R	58.8%		
			Group 3: non-classical mutations without a combination of Del19 or L858R	58.8%		

Abbreviation: EGFR = epidermal growth factor receptor; TKI = tyrosine kinase inhibitor; PR = partial response. ^†^ Case number of the patients receiving an *EGFR* TKI.

**Table A2:** Patients with stage I to III lung cancer harboring rare EGFR mutation.

Patient No.	Age	Sex	Smoking	Initial Stage	TTF-1	EGFR Mutation		TKI Treatment		
						Exon	Mutation	Medication	Line of Treatment	Initial Response
1	68	Female	never	IA	+	exon 21	L861Q	Gefitinib	1st line	PR
2	60	Female	never	IA	+	exon 21	L861Q	Afatinib	1st line	SD
3	42	Male	20 pack-year	IIA	+	exon 18	G719X	Afatinib	1st line	PD
4	72	Male	never	IIIA	+	exon 20	S768I	Erlotinib	2nd line	SD
5	53	Female	never	IIIB	+	exon 18	G724S	Gefitinib	2nd line	PR

Abbreviation: ECOG = Eastern Cooperative Oncology Group; TTF-1 = thyroid transcription factor 1, TTF-1 was often used to confirmed the diagnosis of lung adenocarcinoma.

**Table A3:** Univariate Cox proportional hazards regression analysis to identify the relationship between metastatic sites and survival in patients with stage IV lung cancer harboring rare mutations receiving first-line TKIs.

Metastatic Site	Progression-Free Survival		Overall Survival	
	HR [95% CI]	*p* Value	HR [95% CI]	*p* Value
Brain	1.68 [0.77–3.67]	0.1902	3.82 [1.57–9.26]	0.0030
Leptomeningeal	4.23 [0.97–18.38]	0.0545	15.09 [3.02–75.30]	0.0009
Lung	1.03 [0.56–1.9]	0.9202	0.63 [0.31–1.30]	0.2145
Bone	1.78 [0.97–3.29]	0.0638	1.29 [0.67–2.47]	0.4440
Pleural	1.35 [0.75–2.44]	0.3112	1.26 [0.66–2.39]	0.4816
Pericardial	0.94 [0.33–2.64]	0.9021	1.01 [0.31–3.30]	0.9931
Liver	1.73 [0.75–3.97]	0.1956	1.53 [0.58–4.04]	0.3885
Adrenal	0.5 [0.15–1.66]	0.2565	0.36 [0.08–1.51]	0.1621

Abbreviation: HR = hazard ratio; CI = confidence interval.
